# Hospital Meetings, &c.

**Published:** 1899-12-30

**Authors:** 


### HOSPITAL MEETINGS, &C.

### HOSPITAL FOR SICK CHILDREN.

Mr. Justice Kekewich presided at a special court of governors of the Hospital for Sick Children, Great Ormond Street, on Wednesday, 20th inst., when a resolution authorising Viscount Gort, Mr. Arthur Lucas, Mr. John Murray, and Mr. A. C. Norman, trustees of the hospital, to borrow £24,000 on mortgage of the freehold hospital and the newly-acquired nurses' home adjoining was proposed. The Chairman explained that this was really only a modification of a previous resolution authorising the acquisition of the adjoining land for £30,000. £10,000 of this had been raised, but it had been found necessary to spend some £5,000 on the premises acquired, and they were therefore proposing to borrow £24,000 on mortgage. Mr. Cecil Russell moved the resolution, which was seconded by Mr. Charles Lindo, and carried unanimously.

